# Subjective Well-Being and Bilateral Anterior Insula Functional Connectivity After Exercise Intervention in Older Adults With Mild Cognitive Impairment

**DOI:** 10.3389/fnins.2022.834816

**Published:** 2022-05-10

**Authors:** Junyeon Won, Kristy A. Nielson, J. Carson Smith

**Affiliations:** ^1^Department of Kinesiology, University of Maryland, College Park, MD, United States; ^2^Department of Psychology, Marquette University, Milwaukee, WI, United States; ^3^Department of Neurology, Medical College of Wisconsin, Milwaukee, WI, United States; ^4^Program in Neuroscience and Cognitive Science, University of Maryland, College Park, MD, United States

**Keywords:** MCI (mild cognitive impairment), exercise, subjective well-being, functional connectivity (FC), anterior insula (AI)

## Abstract

While it is well known that exercise training is associated with improvement in subjective well-being among older adults, it is unclear if individuals with cognitive impairment experience the same effects elicited by exercise on subjective well-being. We further explored whether the bilateral anterior insula network may be an underlying neural mechanism for the exercise training-related improvements in subjective well-being. We investigated the effects of exercise training on subjective well-being in older adults (78.4 ± 7.1 years) with mild cognitive impairment (MCI; *n* = 14) and a cognitively normal (CN; *n* = 14) control group. We specifically assessed the relationship between changes in subjective well-being and changes in functional connectivity (FC) with the bilateral anterior insula from before to after exercise training. Cardiorespiratory fitness, subjective well-being, and resting-state fMRI were measured before and after a 12-week moderate-intensity walking intervention. A seed-based correlation analysis was conducted using the bilateral anterior insula as *a priori* seed regions of interest. The associations between bilateral anterior insula FC with other brain regions and subjective well-being were computed before and after exercise training, respectively, and the statistical difference between the correlations (before vs after exercise training) was evaluated. There was a significant Group (MCI vs CN) × Time (before vs after exercise training) interaction for subjective well-being, such that while those with MCI demonstrated significantly increased subjective well-being after exercise training, no changes in subjective well-being were observed in CN. Participants with MCI also showed an exercise training-related increase in the bilateral anterior insula FC. While there was no significant correlation between subjective well-being and bilateral anterior insula FC before exercise training, a positive association between subjective well-being and bilateral anterior insula FC was found in the MCI group after exercise training. Our findings indicate that 12 weeks of exercise training may enhance subjective well-being in older adults diagnosed with MCI and, further, suggest that increased bilateral anterior insula FC with other cortical regions may reflect neural network plasticity associated with exercise training-related improvements in subjective well-being.

## Introduction

Sixteen million American older adults (i.e., approximately 10–20% of the older adult population ≥ 65 years old) are currently living with mild cognitive impairment (MCI) (Langa and Levine, 2014). MCI is presumed to be a prodromal stage of dementia (Bruscoli and Lovestone, 2004), typically characterized by gradual declines in cognition and ability to complete independent activities of daily living (Petersen, 2000). Individuals with MCI can be amnestic, exclusively exhibiting memory impairment, but they typically demonstrate dysfunction in multiple cognitive domains ranging from language, attention, and executive dysfunction, to memory impairment and combinations of these impairments (Saunders and Summers, 2010; Broadhouse et al., 2019; Ding et al., 2019; Won et al., 2021a). Individuals with MCI often also exhibit elevated neuropsychiatric symptoms (Apostolova and Cummings, 2008), which have been associated with their progressive losses of cognition and independence (Albert et al., 1999; Ogawa et al., 2017). Specifically, progressive cognitive impairment has been shown to significantly impact subjective well-being (Dos Santos et al., 2018), a psychological construct that reflects one’s cognitive evaluation of their life satisfaction and affective evaluation of their happiness (Diener, 2000). Subjective well-being is one of the key elements of successful aging (Depp and Jeste, 2006), because low subjective well-being is a strong predictor of adverse health outcomes and death in older adults (Bilotta et al., 2011). Prior investigations in people with MCI have reported that impaired cognitive performance was associated with higher levels of negative affect (Rosenberg et al., 2013; Gates et al., 2014) and poorer psychological well-being compared to cognitively intact elders (Muangpaisan et al., 2008). Therefore, identifying effective interventions that can enhance subjective well-being is an important unmet need for individuals diagnosed with MCI.

Exercise training has well-established positive effects on cardiovascular, muscular, and metabolic health (Gries et al., 2018). Emerging evidence further suggests the salutary effects of exercise training on mental health and subjective well-being. For example, prior meta-analyses reported higher positive affect and life satisfaction (Wiese et al., 2018), self-efficacy (Netz et al., 2005), and happiness (Zhang and Chen, 2019) in those engaging in greater leisure time physical activity. Longitudinal and interventional studies also report that regular participation in exercise is associated with higher levels of subjective well-being and positive affect, as well as fewer symptoms of depression and anxiety in older adults (Camacho et al., 1991; De Moor et al., 2008; Reed and Buck, 2009). Despite this body of evidence, the effects elicited by exercise training on subjective well-being in MCI individuals are not well-understood. To mitigate this gap in the literature, we investigated the effects of a 12-week walking exercise training intervention on subjective well-being in older adults with MCI. We also tested the effects of exercise training on cognitively normal older adults (CN) as a control group. Older adults with MCI demonstrate poorer subjective well-being compared to those with intact cognition (Muangpaisan et al., 2008) and previous work suggests that effects induced by exercise training on cognitive and brain functions were greater in those with MCI than those with intact cognition (Dannhauser et al., 2005; Maquet et al., 2010; Won et al., 2021a). Therefore, we hypothesized that both MCI and CN groups would demonstrate improved subjective well-being in response to exercise training, but the effects of exercise training on subjective well-being would be greater in those with MCI than those in the CN group.

Importantly, the neural foundations of subjective well-being related to ET are unknown. Thus, we sought to address this gap in the literature in healthy older adults and older adults diagnosed with MCI by examining ET-related changes in functional connectivity (FC) within a brain network known to be associated with subjective well-being. Resting-state FC analysis assesses the patterns of functional interaction between remotely located brain regions (Damoiseaux et al., 2006) by using the coherence of functional magnetic resonance imaging (fMRI) blood oxygenation level-dependent (BOLD) measures (Fox et al., 2005). The cingulo-opercular network (i.e., the “salience” network) is one of the key brain networks that detect incoming stimuli, segregate relevant stimuli to guide biobehavioral responses (Menon and Uddin, 2010), and mediate attention shifts between endogenous and exogenous events (Menon, 2010). Importantly, the cingulo-opercular network plays a crucial role in affective processing (Menon and Uddin, 2010) and social well-being (Kong et al., 2016). The bilateral anterior insula and dorsal anterior cingulate cortex are primarily anchored within the cingulo-opercular network, with subcortical nodes including the putamen, ventral striatum, and hypothalamus (Seeley et al., 2007; Sridharan et al., 2008). Among the key brain regions within the cingulo-opercular network, the bilateral anterior insula is linked to interoception, understanding others’ feelings (i.e., emotional awareness), and social well-being (Singer et al., 2009). Indeed, a significant negative correlation has been reported between FC between the left anterior insula and regions within the default mode network and subjective well-being in older adults (Li et al., 2020). Moreover, the anterior insula is responsive to cardiovascular regulation and blood pressure control through afferent feedback, which provides a potential physiological link between exercise and adaptations within the cingulo-opercular network (Kimmerly et al., 2005). Thus, the anterior insula may provide critical neural influence on the effects of exercise training on subjective well-being.

Despite the converging evidence, little is known about the association between anterior insula connectivity and subjective well-being after exercise training. This is particularly important and unstudied in individuals at risk for subjective well-being decline, such as older adults diagnosed with MCI. To address this knowledge gap, the second purpose of this study was to compare the association between the anterior insula FC and subjective well-being before and after the ET intervention. Based on separate pieces of evidence showing both increased intra-salience network connectivity (one of the primary seeds included anterior insula) (Voss et al., 2019) and enhanced subjective well-being (Reed and Buck, 2009) after ET, we hypothesized there would be a positive association between subjective well-being and anterior insula FC, and furthermore, that this correlation would be greater after ET compared to before ET. We further hypothesized that the correlation between anterior insula FC and subjective well-being would be greater after ET in MCI compared to CN.

## Materials and Methods

### Participants

Our prior investigation reported detailed information about the recruitment, eligibility screening, enrollment, and withdrawals (Smith et al., 2013). Briefly, local newspaper advertisements, in-person informational sessions at local retirement communities, and physician referrals were used to recruit participants. We used a structured telephone interview to evaluate preliminary eligibility, including exclusionary health conditions and magnetic resonance imaging (MRI) contraindications for interested individuals. Final eligibility to participate in the present study was determined after participants underwent the informed consent process followed by a neurological assessment (see section “Cognitive Status Evaluation”). The present study was approved by the Institutional Review Board of the Medical College of Wisconsin according to the Helsinki Declaration, and written informed consent was obtained from all participants. Of 407 older adults who responded to study recruitment and advertisements, 92 signed informed consent and underwent neurological examination, 39 started the exercise program, and 35 individuals completed the entire study protocol (17 MCI and 18 CN; ages 60–88). Researchers responsible for data collection sessions were blinded to the participant groupings. All testing sessions occurred within approximately 3–5 days before and after the intervention period.

### Inclusion and Exclusion Criteria

Physically inactive older individuals (i.e., self-rated participation in moderate-intensity physical activity less than 3 days/week in the prior 6 months) were included in the present study. A detailed description about the eligibility criteria and recruitment was previously reported (Smith et al., 2013). Briefly, individuals who reported any of the following were considered ineligible: (1) current or history of neurological or cerebrovascular conditions (e.g., Parkinson’s disease, Huntington’s disease, multiple sclerosis, epilepsy, carotid artery disease, or brain tumor); (2) history of cardiovascular disease or brain injury (e.g., stroke and ischemic attack); (3) MRI contraindications (e.g., metal implants, pacemakers, and claustrophobia); (4) untreated Axis I psychiatric disturbance meeting DSM-IV Axis I criteria, severe depressive symptoms, and substance abuse; (5) >15 score on the 30-item Geriatric Depression Scale (Yesavage, 1988); (6) impairments in independent activities of daily living (Lawton and Brody, 1969); or (7) left-handedness [i.e., <50 of laterality quotient (Oldfield, 1971)].

### Cognitive Status Evaluation

A team of clinical investigators made determinations for each participant’s cognitive status using the core clinical criteria for the diagnosis of MCI by the National Institute of Aging-Alzheimer’s Association criteria for diagnosis of MCI (Albert et al., 2011). The following criteria were utilized to determine the presence of MCI: (1) subjective cognition-related concerns; (2) impaired cognition in more than one cognitive domain; (3) preserved activities of daily living; and (4) absence of dementia. A neurologist evaluated all individuals with probable MCI to exclude other possible causes of cognitive decline (e.g., current clinical depression, history of head trauma, or neurological disease) and to confirm intact activities of daily living and the absence of dementia.

### Subjective Well-Being Assessment

Subjective well-being was assessed using the satisfaction with life scale. The satisfaction with life scale is a short five-item unidimensional measure designed to assess psychometric properties related to satisfaction with life (Pavot et al., 1991). The satisfaction with life scale has been used to measure the life satisfaction component of subjective well-being, reflecting the subjective quality of life and mental health, and it has been predictive of future behaviors such as suicide attempts (Pavot and Diener, 2009). The satisfaction with life scale consists of five statements: “In most ways my life is close to my ideal,” “The conditions of my life are excellent,” “I am satisfied with my life,” “So far I have gotten the important things I want in life,” and “If I could live my life over, I would change almost nothing.” Participants were instructed to indicate their agreement with each item using the scale from 1 (strongly disagree) to 7 (strongly agree). Total scores were calculated across all five statements. Total scores between 5 and 9 indicate extreme dissatisfaction with life, 20 is considered neutral, and scores between 31 and 35 indicate extreme satisfaction with life. The satisfaction with life scale has high internal consistency (Cronbach’s alpha 0.79–0.89) and good test-retest correlations (Cronbach’s alpha 0.80–0.87) (Pavot and Diener, 2008).

### Cardiorespiratory Fitness Test

Participants completed a modified submaximal stress test to assess aerobic fitness before and after the 12-week walking exercise intervention. A modified Balke-Ware protocol (initial exercise speed = 3.2 km/h at 0° grade and grade increase 1°/min) was used on a General Electric (GE) motorized treadmill (Milwaukee, WI, United States). We assessed breath-by-breath measures (expressed as a 15-s average) of ventilation, rate of oxygen (O_2_) consumption, rate of carbon dioxide (CO_2_) production, and the respiratory exchange ratio (RER; CO_2_ production/O_2_ consumption) using a calibrated metabolic measurement system (Parvo Medics, Salt Lake City, UT, United States). The ratings of perceived exertion (RPE) scale (Borg, 1998) was used each minute to monitor perceived exertion during the test. Heart rate (HR) and blood pressure were assessed every 2 min during the test. Heart rate reserve (HRR) was determined based on age-predicted maximal heart rate and resting heart rate measured while seated prior to the exercise test. The test was terminated upon any of the following criteria: (1) reaching 85% of the participant’s maximal HRR; (2) participant’s request to stop the test; or (3) observation of absolute exercise contraindications (e.g., raise in diastolic blood pressure > 110 mmHg). We used linear extrapolation to estimate peak oxygen uptake (V̇O_2peak_), a measure of cardiorespiratory fitness, and at age-predicted maximal heart rate.

### Neuropsychological Test Battery

All participants were assessed with a comprehensive neuropsychological battery before and after the exercise intervention. The neuropsychological test battery evaluated several aspects of cognition including episodic memory, executive function, and processing speed, and a full report can be found in our previous study (Smith et al., 2013). The neuropsychological battery was conducted followed by the cardiorespiratory fitness test, and then an MRI scan on a different day. The battery included the Geriatric Depression Scale (Yesavage, 1988), the Mattis Dementia Rating Scale-2 (Jurica et al., 2001), the Rey Auditory Verbal Learning Test (Rey, 1958), the Logical Memory Test from the Wechsler Memory Scale-III (Wechsler, 1997), the Controlled Oral Word Association Test (COWAT) (Ruff et al., 1996), a semantic fluency test (Tombaugh et al., 1999), and the Clock Drawing Test (Cosentino et al., 2004).

### Walking Exercise Intervention

A 12-week treadmill walking intervention was administered to the study participants. All exercise sessions were administered under the supervision of a certified personal trainer or exercise physiologist. Each exercise session was conducted with no more than two participants. The duration of each exercise session was a total of 50 min including a 30-min exercise session, and 10-min warm-up and cool-down sessions. Each exercise session was administered 4 times/week in local recreation centers. With the goal of achieving a moderate intensity during each session, an HR monitor (Polar Electro, Kempele, Finland) and the RPE scale (Borg, 1998) were used during the intervention to monitor exercise intensity. The exercise session intensity, session duration, and weekly frequency were gradually increased during the first 4 weeks until the participants were walking 30 min per session and the exercise intensity was targeted at 50–60% of HRR during the remaining weeks (i.e., 5–12 weeks). During the first, second, third, and fourth weeks, the participants exercised 2, 3, 3, and 4 times per week at a lower intensity, respectively, and then completed four sessions per week at the target intensity during weeks 5–12 (a total of 44 sessions). The treadmill speed and grade were tailored for each participant in each session using their HR and RPE based on individual baseline exercise capacity. The walking exercise session began with a 10-min warm-up and ended with a 10-min cool-down that consisted of light walking and flexibility exercise. Compliance with the intervention protocol was measured as the proportion of sessions attended. Participants were instructed to not participate in any other forms of structured physical activity during the intervention period.

### Magnetic Resonance Imaging Acquisition

All MRI data were acquired on a 3.0 Tesla GE (Waukesha, WI, United States) MR scanner. A high-resolution T1-weighted anatomical image was acquired for co-registration with the following sequence parameters: 3D Spoiled Gradient Recalled at steady-state protocol (SPGR), field of view (FOV) = 240 mm, voxel size = 0.94 × 0.94 × 1.00 mm, number of excitations (NEX) = 1, slice thickness = 1 mm, repetition time (TR) = 9.6 ms, echo time (TE) = 3.9 ms, inversion recovery preparation time = 450 ms, flip angle = 12°, resolution = 256 × 224, and sequence duration = 6 min. The resting-state BOLD data were acquired using the following sequence parameters: gradient echo planar images, 4.0 mm isotropic voxels, FOV = 240 mm, NEX = 1 mm, slice thickness = 1.0 mm, TR/TE = 2,000/25 ms, axial slices = 36, flip angle = 77°, resolution = 64 × 64, and sequence duration = 6 min. During the resting-state scan, participants were instructed to stay still and to keep their eyes on a fixation cross projected on the screen.

### Structural and Functional Magnetic Resonance Imaging Data Processing

Using FreeSurfer’s (version 5.3.0) automated processing stream (recon-all), the T1-weighted anatomical volumes were processed to generate cortical and subcortical reconstructions based on tissue-specific intensities and atlas probabilities (Fischl, 2012). The resulting inhomogeneity corrected and skull stripped T1-weighted anatomical image was used for the subsequent analyses. To minimize magnetization disequilibrium, Analysis of Functional NeuroImage’s (Cox, 1996) (AFNI, v.17.2.10) 3dTcat function was used to remove the first three volumes of the functional image time-series. The truncated time-series were then realigned using Slice-Oriented Motion Correction (SLOMOCO) using both in- and out-of-plane motion parameters to correct for misalignment between consecutive slices (Beall and Lowe, 2014). Next, using AFNI’s align_epi_anat function, the motion-corrected functional volumes were co-registered to FreeSurfer-rendered anatomical images and were visually inspected for proper alignment and no further correction was administered. The co-registered time-series were subsequently submitted to AFNI’s single-subject preprocessing stream (proc.py). Volumes with outlier fraction threshold (>10%) were removed from the time-series to attenuate the spurious effects of head motion. AFNI’s 3dDespike and 3dTshift functions were used to de-spike the remaining volumes to reduce high-intensity transients within the BOLD signal and to time-shift to the beginning of the TR, respectively. Next, using AFNI’s non-linear transformation (3dQwarp) function, the anatomical image and anatomical followers (FreeSurfer-processed gray matter, white matter, and ventricular segmentations) were warped to 2 mm MNI standard space (AFNI’s MNI152_T1_2009c template). Using the artifact correction method ANATICOR (which anatomically models signals), nuisance physiological artifacts (e.g., motion derivatives and signals from ventricles and white matter) were removed to further exclude local and global artifacts (Jo et al., 2013). There was no statistical difference in the percentage of removed TRs between the before (0.88 ± 0.28%) and after exercise training scans (0.98 ± 0.35%) (*p* = 0.062; paired *t*-test). We also did not find significant differences in motion between groups (*p* = 0.142; MCI group before exercise training: 1.00 ± 0.03% after exercise training 1.00 ± 0.04%; CN group before exercise training 0.07 ± 0.01% after exercise training 0.09 ± 0.02%; repeated-measures ANOVA), and there was not a significant Time × Group interaction (*p* = 0.077; repeated-measures ANOVA) on removed TRs. Thus, the effects of head movement were not included as a covariate in the fMRI data analysis.

### Functional Connectivity Analysis

**FIGURE 1:**
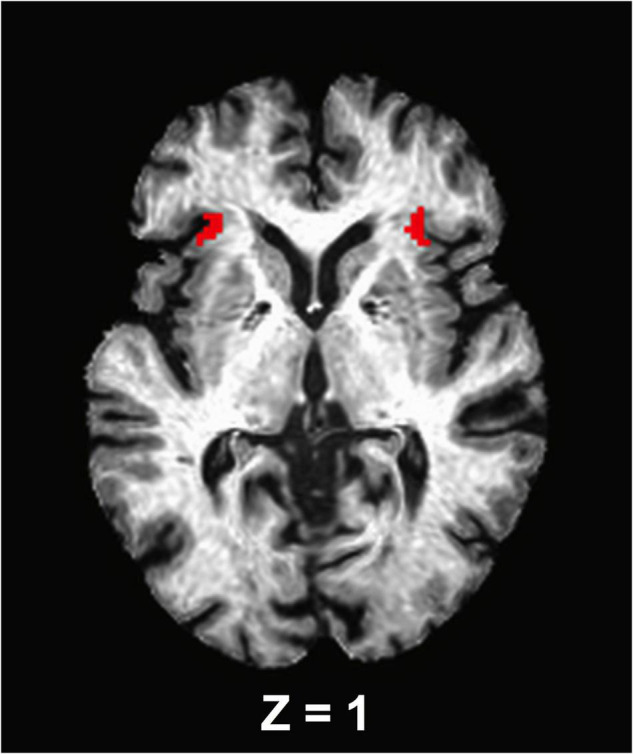
An axial view of the anterior insula seed regions. The seed masks are exhibited on a representative subject’s structural image.

A seed-based correlation analysis was used to assess the anterior insula functional connectivity and thus, the bilateral anterior insula were selected as *a priori* seed regions of interest. To accomplish this, left and right anterior insula masks [anterior segment of the circular insular from the Destrieux cortical atlas, which is based on a parcellation scheme using the division of the cortex into gyral and sulcal regions (Destrieux et al., 2010)] were extracted from each participant’s Freesurfer-processed cortical parcellation anatomical follower that was warped to the standard MNI space (Figure 1). Using AFNI’s 3dcalc function, the bilateral insula masks were merged. The final segmentations for each seed were visually inspected for quality assurance. No further correction was administered. The average signal time series from the bilateral insula were then extracted from the seed regions and cross-correlated with all voxels in the brain to isolate an FC brain map. These maps were created for each participant and each experimental time point (e.g., before and after ET). Finally, correlation coefficients were standardized for group-level analysis using a Fisher’s *r*-to-*z* transformation (Fisher, 1915).

### Gray Matter Voxel-Wise Group Analysis

Based on FreeSurfer segmentation, the whole-brain gray matter mask in which white matter and ventricles are excluded was created for each participant. By combining the gray matter mask across participants using AFNI’s 3dcalc function, a group-level mask was created. Subject-level *z*-scored correlation maps were submitted to AFNI’s linear mixed-effects model (3dLME) to investigate (1) changes in the bilateral anterior insula FC from before to after ET (i.e., main effects of Time) across participants; (2) differences between groups (MCI vs CN before exercise training and after exercise training); and (3) Group (MCI vs CN) × Time (before vs after ET) interaction. AFNI’s cluster-size threshold computation program (3dClustSim) was used to control the whole brain family-wise error rate (FWER) at *p* < 0.05 based on a voxel-wise probability threshold of *p* < 0.001 and minimum cluster size of *k* ≥ 41 (328 mm^3^).

### Statistical Analysis

We first determined normality using the Shapiro–Wilk test. The baseline demographic differences between groups (MCI vs CN) were evaluated using independent sample *t*-tests (or Wilcoxon signed-rank tests for non-parametric data) or Fisher’s exact test for discrete data [e.g., number of female participants or apolipoprotein E epsilon-4 allele (APOE-ε4) carrier]. Normality of satisfaction with life scale and the geriatric depression scale (GDS) were also computed using the Shapiro–Wilk test. We used repeated-measures ANOVA to test the main effects of Time (i.e., before vs after ET), Group (i.e., MCI vs CN), and Group × Time interaction on the satisfaction with life scale score and GDS. We also tested the main effects of Time within each group. The association between the satisfaction with life scale score and anterior insula FC was subsequently investigated before and after ET, respectively. To accomplish this, partial correlation analysis was used. Prior to this test, we conducted bivariate correlation tests to evaluate relationships between age and variables of interest (satisfaction with life scale and bilateral anterior insula FC). There were no significant correlations between age and variables of interest (*p* ≥ 0.199). We did not find significant correlations between sex and variables of interest (*p* ≥ 0.104). Furthermore, there were no significant correlations between motion metric and variables of interest (*p* ≥ 0.497). Therefore, the correlation analyses were unadjusted. After computing the correlation between satisfaction with life scale score and anterior insula FC before and after ET, respectively, the statistical difference between correlation (i.e., before ET: satisfaction with life scale-bilateral anterior insula FC vs after ET: satisfaction with life scale-bilateral anterior insula FC) was computed. The “cocor” (a comprehensive solution for the statistical comparison of correlations model) package within R software (Diedenhofen and Musch, 2015) was used to assess the statistical difference between the satisfaction with life scale-bilateral anterior insula FC correlation as a function of Time. All other statistical tests were performed using SPSS (v. 26.0, IBM, Armonk, NY, United States). Statistical significance was determined at alpha = 0.05 for all tests.

## Results

### Demographic Characteristics

Of the 35 participants, 7 individuals (3 MCI and 4 CN) were excluded from analysis due to missing satisfaction with life scale data. The remaining 28 participants were included in the final analyses (14 MCI and 14 CN). Data were missing for some participants for V̇O_2peak_ (1 CN, 2 MCI), and GDS (2 CN, 1 MCI); these participants are included in all analyses not involving these variables. The demographic data for the 28 participants are shown in Table 1. Participants had an average age of 78.4 years and education of 15.7 years. 78.5% were women and there were 10 APOE-ε4 carriers. There were no significant differences between the MCI and CN groups in the baseline demographic characteristics including age, sex, education, number of APOE-ε4 carriers, V̇O_2peak_, and Lawton Instrumental Activities of Daily Living (Table 1).

**TABLE 1:** Demographic information for study participants.

	Total sample (*n* = 28)	MCI (*n* = 14)	CN (*n* = 14)	Group differences
	
	Mean ± SD	Mean ± SD	Mean ± SD	*p*-value
**Demographics**				
Age (years)	78.4 ± 7.1	80.8 ± 5.8	76.0 ± 7.6	0.074
Female (*n*, %)	22 (78.5%)	9 (64.2%)	13 (92.8%)	0.065_F_
Education (years)	15.7 ± 2.2	15.0 ± 2.2	16.4 ± 2.1	0.097
APOE-ε4 carriers (*n*, %)	10 (35.7%)	5 (35.7%)	5 (35.7%)	1.000_F_
**Cardiorespiratory fitness**				
Baseline V̇O_2peak_ (ml/kg/min)	18.8 ± 4.4	18.0 ± 3.7	19.7 ± 5.0	0.321
**Activities of daily living**				
Baseline Lawton ADL	4.8 ± 0.3	4.8 ± 0.3	4.7 ± 0.4	0.636

*MCI, mild cognitive impairment; CN, normal cognition control;_F_, Fisher’s Exact Test; APOE-ε4, apolipoprotein E epsilon 4 allele; V̇O_2peak_, peak rate of oxygen consumption; DRS-2, Mattis dementia rating scale-2; ADL, activities of daily living.*

### Aerobic Fitness, Subjective Well-Being, Geriatric Depression Scale, and Cognitive Function

**FIGURE 2:**
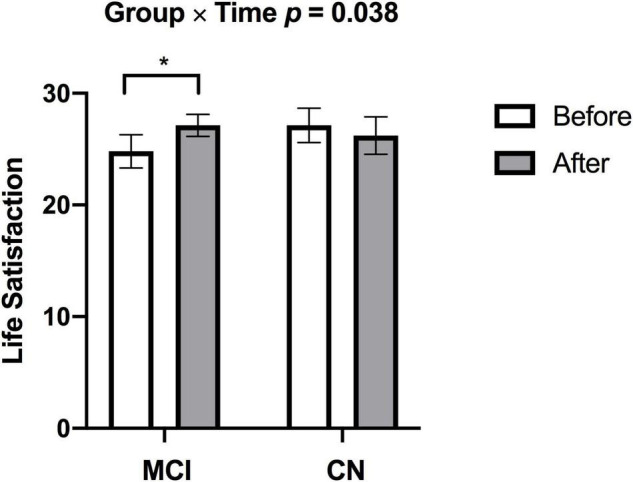
Individuals with MCI had lower subjective well-being at baseline but demonstrated increased subjective well-being after exercise training (*p = 0.003). There was no change in subjective well-being in CN after exercise training. *p-*values above bar graphs indicate the Group × Time interaction.

There was a significant increase in V̇O_2peak_ from before to after ET in both groups (missing data: 1 CN, 2 MCI) [*F*_(1,23)_ = 5.887, *p* = 0.023, η^2^_p_ = 0.204]. There was a significant Group × Time [*F*_(1,26)_ = 4.770, *p* = 0.038, η^2^_p_ = 0.155] for satisfaction with life scale data (see Figure 2), such that subjective well-being significantly increased in participants with MCI (*p* = 0.003), but no subjective well-being change occurred in the CN group after ET (*p* = 0.382). Yet, no main effects of Time [*F*_(1,26)_ = 0.266, *p* = 0.610, η^2^_p_ = 0.010] and Group [*F*_(1,27)_ = 0.324, *p* = 0.574, η^2^_p_ = 0.012] were found in satisfaction with life scale. While there was a significant main effect of Group for the GDS [*F*_(1,23)_ = 7.247, *p* = 0.013, η^2^_p_ = 0.240] indicating higher depression scores in the MCI, there were no significant effects of Time [*F*_(1,23)_ = 0.015, *p* = 0.904, η^2^_p_ = 0.001] or Group × Time [*F*_(1,23)_ = 0.567, *p* = 0.459, η^2^_p_ = 0.024] (missing data 2 CN, 1 MCI). There were group differences (CN > MCI), as well as significant Time main effects and one Time by Group interaction effect on multiple cognitive tasks. See Table 2 for details.

**TABLE 2:** Cardiorespiratory fitness, life satisfaction, GDS, and cognitive function data for study participants.

	Total Sample (*n* = 28)		MCI (*n* = 14)		CN (*n* = 14)		Time	Group	Group × Time
	Before	After	Before	After	Before	After			
	
	Mean ± SD	Mean ± SD	Mean ± SD	Mean ± SD	Mean ± SD	Mean ± SD	*p*-value (η^2^_p_)	*p*-value (η^2^_p_)	*p*-value (η^2^_p_)
**Cardiorespiratory fitness**									
V̇O_2peak_ (ml/kg/min)	18.8 ± 4.4	20.5 ± 3.7	18.4 ± 3.8	20.6 ± 3.2	19.7 ± 5.0	20.7 ± 4.2	**0.023 (0.203)**	0.649 (0.009)	0.334 (0.040)
**Subjective well-being**									
Life satisfaction scale	26.1 ± 6.0	26.6 ± 5.3	24.6 ± 5.7	27.0 ± 4.2	27.7 ± 6.1	26.2 ± 6.3	0.610 (0.010)	0.574 (0.012)	**0.038 (0.155)**
**Depression**									
GDS	4.8 ± 3.1	4.6 ± 3.3	6.4 ± 3.6	5.7 ± 2.7	3.4 ± 1.3	3.9 ± 3.6	0.904 (0.0006)	**0.013 (0.239)**	0.459 (0.024)
**Cognitive function**									
DRS-2 total	35.7 ± 1.3	35.9 ± 1.2	35.3 ± 1.7	35.8 ± 1.0	36.2 ± 0.8	36.0 ± 1.4	0.583 (0.012)	0.171 (0.071)	0.327 (0.037)
RAVLT trial 1–5	43.4 ± 13.7	45.4 ± 14.6	37.2 ± 13.7	39.6 ± 15.8	50.1 ± 10.3	51.7 ± 10.4	0.221 (0.059)	**0.013 (0.221)**	0.803 (0.003)
RAVLT immediate recall	8.6 ± 4.3	9.1 ± 4.2	6.5 ± 4.1	7.2 ± 4.7	10.9 ± 3.5	11.1 ± 2.3	0.234 (0.056)	**0.008 (0.251)**	0.538 (0.015)
RAVLT delayed recall	8.3 ± 4.6	8.8 ± 4.5	6.3 ± 4.5	6.7 ± 4.8	10.5 ± 3.9	11.1 ± 3.1	0.407 (0.028)	**0.008 (0.249)**	0.825 (0.002)
LM immediate recall	36.1 ± 13.3	39.6 ± 13.5	29.0 ± 13.6	33.3 ± 16.4	43.5 ± 7.7	46.1 ± 4.1	**0.007 (0.259)**	**0.004 (0.283)**	0.330 (0.038)
LM delayed recall	22.2 ± 10.0	24.3 ± 10.3	17.4 ± 10.5	19.4 ± 11.2	27.4 ± 6.3	29.6 ± 6.1	0.061 (0.134)	**0.005 (0.274)**	0.934 (0.0002)
COWAT	38.4 ± 11.1	41.9 ± 12.2	34.9 ± 11.8	41.6 ± 13.8	41.6 ± 9.8	42.2 ± 11.0	**0.014 (0.234)**	0.425 (0.028)	**0.037 (0.175)**
Semantic fluency	17.3 ± 6.8	17.6 ± 8.1	14.2 ± 7.4	13.7 ± 8.8	20.4 ± 4.5	21.4 ± 5.2	0.765 (0.004)	**0.007 (0.247)**	0.456 (0.022)
Clock drawing	2.0 ± 1.1	1.7 ± 0.8	2.5 ± 1.0	2.1 ± 0.8	1.5 ± 1.0	1.2 ± 0.7	0.050 (0.140)	**0.005 (0.262)**	0.499 (0.018)

*MCI, Mild cognitive impairment; CN, normal cognition control; V̇O_2peak_, peak rate of oxygen consumption; GDS, geriatric depression scale; DRS-2, Mattis dementia rating scale-2; RAVLT T1-5, Rey auditory verbal learning test; LM, logical memory task; COWAT, controlled oral word association test; p-values and effect size (η^2^_p_) reflect the Time, Group, and Group × Time effects from repeated-measures ANOVA. Bold indicates p < 0.05.*

### Voxel-Wise Bilateral Anterior Insula Functional Connectivity

**FIGURE 3:**
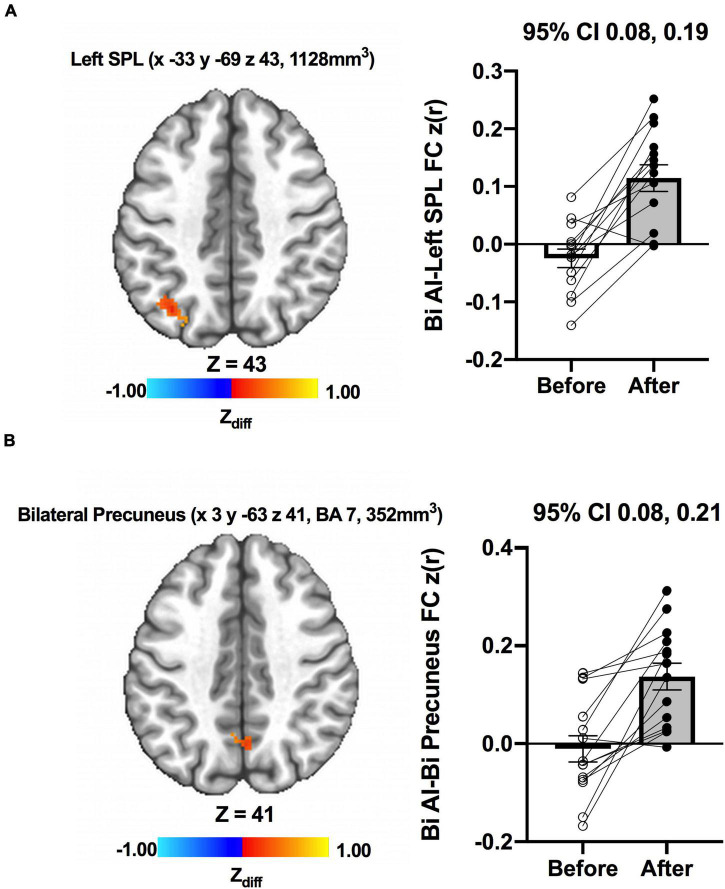
Increases in functional connectivity between the anterior insula and **(A)** left superior parietal lobe, inferior parietal lobe, and **(B)** bilateral precuneus were found after exercise training in MCI individuals. Adjacent bar graphs indicate the connectivity between the anterior insula and respective regions (±SEM) before and after exercise training. 95% CI above bar graphs indicates 95% confidence interval in the difference from before to after exercise training.

**FIGURE 4:**
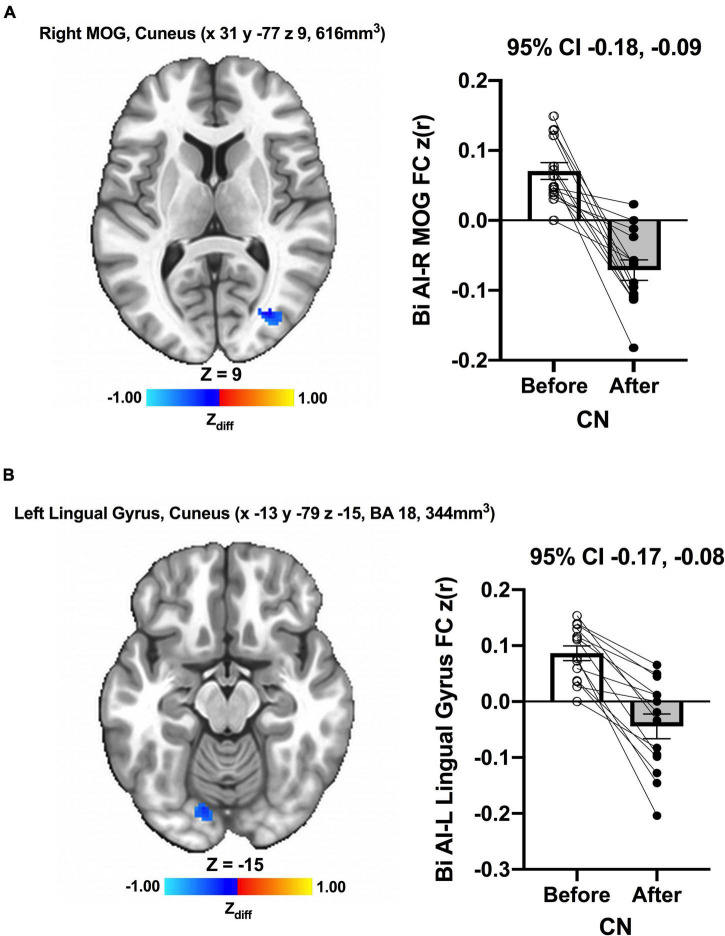
Decreases in functional connectivity between the anterior insula and **(A)** right middle occipital gyrus and cuneus, and **(B)** left lingula gyrus and cuneus were found after exercise training in CN individuals. Adjacent bar graphs indicate the connectivity between the anterior insula and respective regions (± SEM) for before and after exercise training. 95% CI above bar graphs indicates 95% confidence interval in the difference from before to after exercise training.

**FIGURE 5:**
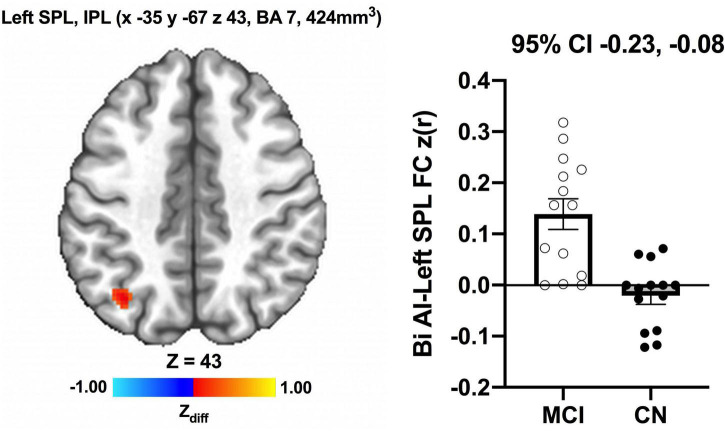
Greater functional connectivity between the anterior insula and left superior parietal lobule and inferior parietal lobule were found after exercise training in individuals with MCI compared to individuals with CN. Adjacent bar graphs indicate the connectivity between the anterior insula and respective regions (±SEM) for after exercise training. 95% CI above bar graphs indicate 95% confidence interval in the group difference (CN vs MCI).

We detected no significant main effects of Time, Group, and Group × Time interaction effects on the bilateral anterior insula FC. Also, there were no significant baseline differences in FC between groups. However, significant increase in FC between the bilateral anterior insula and clusters within left superior parietal lobule, inferior right precentral gyrus, and precuneus [MNI −33 −69 43 (LPI), BA 7, 19, 1,128 mm^3^] was found in the MCI group (Figure 3; Panel A). There was also a significantly increased connectivity between the bilateral anterior insula and bilateral precuneus [MNI 3 −63 41 (LPI), BA 7, 352 mm^3^] in individuals with MCI (Figure 3; Panel B). In the CN group, there was decreased FC after exercise training compared to before exercise training between bilateral insula clusters and clusters within the right middle occipital gyrus and cuneus [MNI 31 −77 9 (LPI), 616 mm^3^] (Figure 4; Panel A) with the left lingual gyrus and cuneus [MNI −13 −79 −15 (LPI), BA 18, 344 mm^3^] (Figure 4; Panel B). Finally, while there were no significant group differences at the baseline, individuals with MCI, compared to their CN counterparts, demonstrated a greater FC between the bilateral anterior insula and clusters within the superior parietal lobule and inferior parietal lobule [MNI −35 −67 43 (LPI), BA 7, 424 mm^3^] after ET (Figure 5).

### Associations Between Subjective Well-Being and Bilateral Anterior Insula Functional Connectivity

**FIGURE 6:**
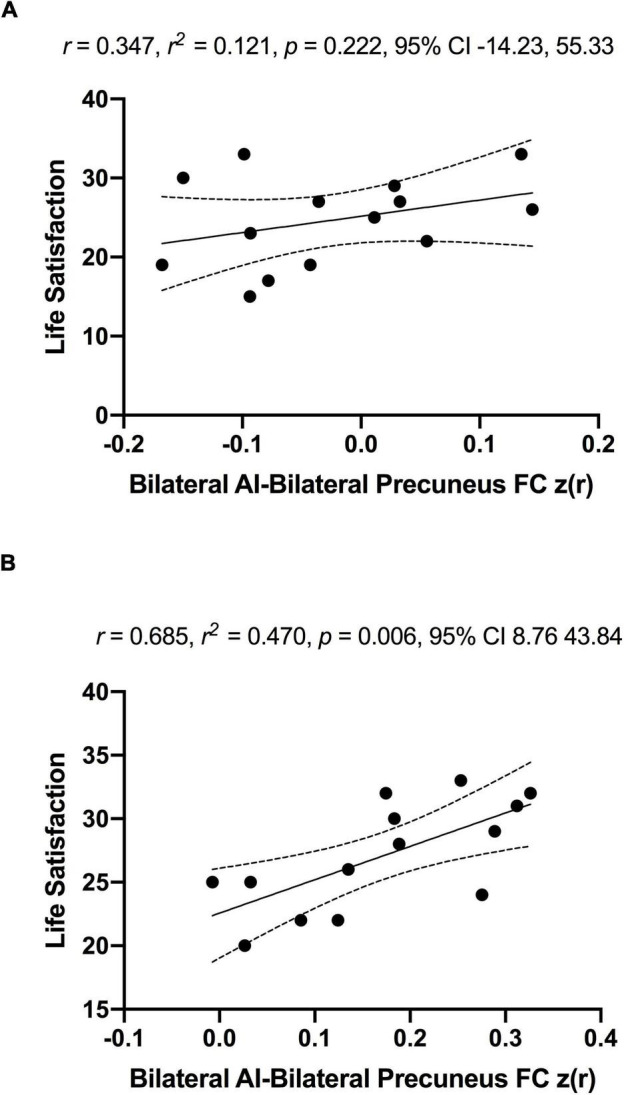
No significant associations between anterior insula-FC and life satisfaction scale in MCI individuals before (Panel **A**) and significantly positive association after exercise training (Panel **B**). The *r*, *r*^2^, *p*-value, and 95% confidence interval reflect the correlation of anterior insula-FC and life satisfaction and dotted curves indicate 95% confidence interval around the regression line.

As a significant change in subjective well-being was only found in the MCI group (Before ET: 24.6 ± 5.7, After ET: 27.0 ± 4.2; *p* = 0.003), the association between subjective well-being and bilateral anterior insula FC was only assessed in the MCI group. To examine the association between subjective well-being and bilateral anterior insula FC, average *z*-scored connectivity within the regions shown in Figure 3 was extracted from subject-level correlation maps. First, there were no significant correlations between subjective well-being and bilateral anterior insula FC with left SPL, IPL, and precuneus both before [*R* = 0.123, *R^2^* = 0.015, *p* = 0.675] and after ET [*R* = 0.007, *R^2^* = 0.00005, *p* = 0.980]. While there was no significant correlation between the subjective well-being and bilateral anterior insula FC with bilateral precuneus before ET [*R* = 0.347, *R^2^* = 0.121, *p* = 0.222] (Figure 6; Panel A), a significantly positive correlation was observed after ET [*R* = 0.685, *R^2^* = 0.470, *p* = 0.006] (Figure 6; Panel B). Therefore, correlations between subjective well-being and bilateral anterior insula-bilateral precuneus FC were used for the subsequent analysis. Lastly, we compared the statistical difference in the correlations between subjective well-being and bilateral anterior insula-bilateral precuneus FC before and after ET. Results revealed that there was a significant difference between the correlations (*Z* = −4.862, *p* < 0.001), suggesting that the correlation between subjective well-being and bilateral anterior insula-bilateral precuneus FC became significantly different after ET compared to before ET in participants with MCI.

## Discussion

The present study investigated the effects of a 12-week walking intervention on subjective well-being and bilateral anterior insula FC in older adults with MCI and normal cognition. The walking exercise intervention resulted in an approximately 10% increase in cardiorespiratory fitness (V̇O_2peak_) across participants. Those with MCI had lower subjective well-being at baseline, which increased to equal that of the CN group over the 12 weeks; CN older adults maintained subjective well-being over the 12 weeks. Moreover, opposite patterns of anterior insula FC were found between the groups after ET, such that there was an ET-induced increase in the FC between the bilateral anterior insula and other brain regions in MCI, but there was decreased FC between the bilateral anterior insula and other brain regions in the CN group. Finally, in the MCI group, we observed a positive association between anterior insula FC with the bilateral precuneus and subjective well-being after exercise training; an association that was not observed before ET.

Previous aging literature suggests enhanced subjective well-being in older adults after exercise training. For example, a 4-month physical activity program consisting of aerobic, strength, and balance training demonstrated enhanced subjective well-being in older adults (67–85 years old) (Delle Fave et al., 2018). According to Mihalko and McAuley (1996), older adults (83 years) who completed an 8-week upper-body high-intensity strength training demonstrated significantly greater increases in muscular strength and corresponding increase in levels of subjective well-being (Mihalko and McAuley, 1996). Similarly, there was a significantly improved subjective well-being following a 32-week aerobic exercise and light stretching intervention compared to a health education control condition in older adults (65 years) (McMurdo and Burnett, 1992). Lastly, Brawley et al. (2000) found significantly enhanced subjective well-being in response to a 12-week aerobic exercise program (Brawley et al., 2000). The present results extend the existing body of literature by suggesting the salutary effects of exercise training on subjective well-being also occur in older adults who live with cognitive decline.

Previous investigations consistently reported a lower subjective well-being in people with MCI compared to CN older adults (St. John and Montgomery, 2010; Dos Santos et al., 2018). Although it was not statistically significant, we observed a trend toward lower baseline subjective well-being in the MCI group compared to the CN group (MCI 24.6 ± 5.7 vs CN 27.7 ± 6.1; *p* = 0.183). One possible factor driving the lack of ET-related changes in subjective well-being among the CN group could be a relatively high baseline subjective well-being. That is, the CN group had higher subjective well-being than the MCI group at baseline, indicating a possible ceiling effect that would not permit increased scores in the CN group but would allow greater opportunity for the participants with MCI to experience ET-related enhancement in subjective well-being. Indeed, exercise is likely to elicit greater effects on aspects of health-related quality of life among individuals experiencing age/pathology-related decline in brain function (Rippe, 2019). It is also important to note that our exercise intervention brought the subjective well-being of participants with MCI up to the level of the CN older adults at the end of the study. Nevertheless, this must be interpreted with caution due to the small sample size in the present study and the lack of expected change in the CN group. Therefore, the possible differential effects of ET on subjective well-being based on cognitive status in older adults should be further clarified in the future using a larger sample.

A novel finding of the present study was that ET resulted in increased connectivity between a hub of the cingulo-opercular network and regions of the parietal cortex among participants with MCI. In support of the increased anterior insula FC in the present study, 6-month aerobic exercise training, in addition to regular intake of nutritional supplement, led to increased anterior insula FC compared to the baseline in older adults (64.6 years) (Voss et al., 2019). Furthermore, in older adults with MCI, a 24-week aerobic exercise training intervention was associated with changes in regional glucose metabolism within the areas of the cingulo-opercular network, possibly reflecting improved function and neuroplasticity within the network (Porto et al., 2018). It has been suggested that regular participation in exercise promotes elevated functional connectivity between brain regions in the aging brain (Voss et al., 2010). Indeed, the exercise training-induced increase in FC among those with MCI is consistent with our past work that demonstrated increased default-mode network (DMN) (Chirles et al., 2017), cerebellar network (Won et al., 2021a), and hippocampal FC (Won et al., 2021b) in response to exercise training. Hence, our results show that exercise training-related increased network connectivity aligns well with the prior exercise neuroimaging evidence in participants with MCI.

Notably, the correlation between the anterior insula-precuneus connectivity and subjective well-being became significantly positive after ET in the MCI group. The relationship between anterior insula FC and subjective well-being presented in this study is supported by the work of Li et al. (2020) who reported an association between lower dorsal anterior insula FC and higher subjective well-being in older adults (70.6 years) (Li et al., 2020). Li et al. (2020) demonstrated that the brain regions that were functionally connected to the dorsal anterior insula were centered on core hubs of the default-mode network (DMN) regions, including the anterior medial prefrontal cortex and inferior parietal lobe. Although the direction of the association between the bilateral anterior insula FC and subjective well-being was opposite, presumably due to ET effects, we also observed that the relationship between the bilateral anterior insula functional network and subjective well-being was specific to the core hub region of the DMN (i.e., precuneus) (Buckner et al., 2008). The precuneus is a crucial region for integrating different types of information from other brain regions, and greater right precuneus volume has been associated with a greater subjective happiness score (Sato et al., 2015). In support, there is evidence for greater precuneus gray matter volume to be associated with greater satisfaction with life (Kong et al., 2015) and higher subjective happiness (Sato et al., 2015). Consistent with the role of the precuneus, the insula, seed region for the present study, is one of the most frequently reported regions that is essential for processing emotional awareness and subjective feelings including subjective well-being (Craig, 2002). Neuroimaging studies suggest that insula activation is linked to remembering happy events (Suardi et al., 2016), emotional and affective process (Uddin, 2015), and is positively correlated with ratings of subjective well-being (Rutledge et al., 2014). Moreover, larger insular cortex volume is associated with greater psychological well-being (Lewis et al., 2014). This line of evidence corroborates our finding that increased connectivity between the insula and precuneus may constitute a unique neural basis for improved subjective well-being after exercise training in older adults diagnosed with MCI.

### Potential Mechanisms

Although the neurophysiological mechanisms underpinning the ET-related increase in FC among individuals with MCI are not completely understood, consistent exercise is associated with increased skeletal muscle capillarization and enhanced mitochondrial function in the brain (Kayes and Hatfield, 2019). This ET-induced enhancement in mitochondrial function and mitochondrial density in the brain is instrumental for long-term potentiation (Bettio et al., 2019) and synaptogenesis (Steib et al., 2014). This neural adaptation to exercise training may promote neuronal signaling, proliferation of new neurons into brain networks, and, in turn, strengthen functional network integrity (Voss et al., 2016; Won et al., 2021c). For individuals diagnosed with MCI, who may have reached a critical threshold for age- and pathology-related neural changes (Barulli and Stern, 2013), exercise training-related adaptations may facilitate a stronger “neural scaffolding,” presumably through an adaptive compensatory response (i.e., recruitment of additional neural resources) (Reuter-Lorenz and Park, 2014; Won et al., 2019; Won et al., 2020). Although the need to mount a compensatory response in old age is frequently associated with cognitive decline, extensive neural compensation in the aging brain has also been shown to support sustained effective brain function (Rao et al., 2015). Therefore, increased anterior insula FC may reflect beneficial compensatory responses induced by exercise training in individuals with MCI, which was also supported by its positive correlation with subjective well-being. Conversely, there was a decrease in anterior insula FC in response to exercise training in cognitively intact older adults in the present study. In our previous investigation using the same participants, there was an exercise training-elicited increase in default mode network FC in the participants with MCI, and a decrease in default mode network FC in the CN group (Chirles et al., 2017). Thus, both the current and previous study (Chirles et al., 2017) suggest that exercise training may result in differential responses on the functional networks of the brain depending on cognitive status in older individuals. Decreased FC after exercise training in cognitively intact older adults may be associated with increased neural reserve (or efficiency) of the functional network or a shift in allocation of resources or attentional focus (Smith et al., 2013). However, this hypothesis remains speculative and should be further investigated.

Another possible mechanism involves changes in cerebrovascular reactivity, especially due to the location of the anterior insula and middle cerebral artery. The main arterial source for the insula is the M2 segment of the middle cerebral artery (Türe et al., 2000), which has been shown to increase cerebrovascular reactivity in response to exercise training (Murrell et al., 2013). We were not able to measure cerebrovascular reactivity in our study. However, our previous report using the same cohort found that exercise training-related reductions in cerebral blood flow within the left insula region were associated with improved verbal fluency performance (Alfini et al., 2019), which supports the speculation that changes in cerebrovascular reactivity may be associated with changes in the anterior insula FC after exercise training. Future studies need to clarify the association between cerebrovascular reactivity and functional connectivity to better understand the underlying mechanism of change induced by long-term exercise.

### Strengths and Limitations

A major strength of the present study was the well-attended (compliance rate of ∼96%) and well-supervised intervention, which may have driven the increase in cardiorespiratory fitness after ET (10.5% increase in V̇O_2peak_). Furthermore, the psychological benefits of ET were examined in older adults with an objective diagnosis of MCI, who have been understudied. Despite these strengths, the present study should be interpreted with caution until it is replicated in a larger randomized controlled trial due to the lack of a non-exercise (or active) control group. Next, we used a relatively short intervention (i.e., 12 weeks); thus, we may have missed potential effects that could manifest after a longer exercise intervention (e.g., 6–12 months). Nevertheless, the present study suggests that ET-related effects on subjective well-being and anterior insula FC may emerge within 3 months. Thus, it may be useful for longer interventions to document the time course of these effects in addition to measurements before and after the intervention. In addition, the present study employed a relatively small sample (*n* = 28) with homogeneous demographic characteristics [e.g., predominantly women (78%) and highly educated (average of 15 years)]. Therefore, our results may not be generalizable to the entire older adult population. An important additional direction for future research is to recruit a larger sample with diverse fitness levels to further assess the effects of exercise training on subjective well-being in individuals with MCI. Lastly, the ceiling effect may have occurred in the subject well-being scale for the CN group, which may have impeded capturing the intervention effect in CN older adults.

## Conclusion

In conclusion, a 12-week walking intervention improved subjective well-being in older adults with impaired cognition, which occurred independently of changes in cognitive function. Hence, the present study suggests that improvement in subjective well-being after exercise training may be an independent construct from cognition in older adults with MCI. Our results also suggest that the increase in FC between the anterior insula and precuneus may be an important neural basis for the ET-induced enhancements in subjective well-being among older adults diagnosed with MCI. Consistent with our previous studies using the same MCI cohort that showed the ET-related effects were evident in the insula (Chirles et al., 2017; Alfini et al., 2019; Callow et al., 2021), our work collectively suggests that the insula is a brain region that is particularly impacted by ET in individuals with MCI. While most MCI studies have focused on improvement in cognitive function, the present study extends the existing literature by suggesting that the neuroprotective effects induced by exercise may be extended to the psychological well-being of older adults with MCI. Older adults with MCI typically demonstrate lower subjective well-being compared to their cognitively normal counterparts, which could be a strong predictor of adverse health outcomes and death. The present study, therefore, conveys an important public health message that a lifestyle intervention as simple as moderate-intensity walking may result in neural network plasticity that is related to improved quality of life in older adults facing possible progression to AD.

## Data Availability Statement

The raw data supporting the conclusions of this article will be made available by the authors, without undue reservation.

## Ethics Statement

The studies involving human participants were reviewed and approved by the Medical College of Wisconsin. The patients/participants provided their written informed consent to participate in this study.

## Author Contributions

JW and JS developed the study idea and collectively developed the analytic strategy. JS and KN developed the overall study protocol and collected data. JW processed and analyzed imaging data, drafted the manuscript, and created the figures. All authors interpreted the data, edited the manuscript, reviewed, revised, and approved the final manuscript.

## Author Disclaimer

The content is solely the responsibility of the authors and does not necessarily represent the official views of the NIH.

## Conflict of Interest

The authors declare that the research was conducted in the absence of any commercial or financial relationships that could be construed as a potential conflict of interest.

## Publisher’s Note

All claims expressed in this article are solely those of the authors and do not necessarily represent those of their affiliated organizations, or those of the publisher, the editors and the reviewers. Any product that may be evaluated in this article, or claim that may be made by its manufacturer, is not guaranteed or endorsed by the publisher.
